# How Can We Introduce ART into Wild Felid Conservation in Practice? Joint Experience in Semen Collection from Captive Wild Felids in Europe

**DOI:** 10.3390/ani12070871

**Published:** 2022-03-30

**Authors:** Sylwia Prochowska, Wojciech Niżański, Feline Snoeck, Eline Wydooghe, Ann Van Soom, Joanna Kochan, Vasyl Stefanyk

**Affiliations:** 1 Wrocław University of Environmental and Life Sciences, 50-375 Wrocław, Poland; 2 Department of Internal Medicine, Reproduction and Population Medicine, Ghent University, 9820 Merelbeke, Belgium; felinesnoeck@gmail.com (F.S.); eline.wydooghe@vives.be (E.W.); ann.vansoom@ugent.be (A.V.S.); 3 Department of Animal Reproduction, Anatomy and Genomics, University of Agriculture in Kraków, 30-059 Krakow, Poland; joanna.kochan@urk.edu.pl; 4 Department of Obstetrics, Gynecology and Biotechnology of Animal Reproduction Named after H.V.Zvereva, Stepan Gzhytskyi National University of Veterynary Medicine and Biotehnologies Lviv, 79010 Lviv, Ukraine; stefanyk@bigmir.net

**Keywords:** wild felids, semen, artificial reproductive techniques, infertility

## Abstract

**Simple Summary:**

Artificial reproductive techniques (ART), such as cryopreservation of sperm cells and artificial insemination, are useful tools for species conservation. However, there is relatively little information published about their introduction into clinical practice for wild felids. The aim of this paper was to describe how those techniques were applied by three European teams in various species of wild felids. In total, this article presents 22 semen collection attempts in 12 species of wild felids, 15 of which were successful and resulted in the collection of at least one million spermatozoa. The failures in obtaining spermatozoa were most probably due to (1) male infertility, (2) wrong age/non-breeding season, or (3) recent multiple copulations. The cases presented in the article confirm that although ART have been introduced into clinical practice, they are mostly used in cases of infertility, not as routine breeding tools. Greater involvement of zoological gardens and private breeders is required, as many chances for preservation of valuable material are lost.

**Abstract:**

Although artificial reproductive techniques (ART) are considered to be a valuable tool for species conservation, information about their introduction into clinical practice for wild felids is limited. The aim of this paper was to jointly describe cases of non-experimental sperm collection from males of various species of wild felids, performed by three European centers focused on feline reproduction. In total, the article presents 22 attempts of semen collection in 12 species of wild felids. The reasons for semen collection were: fertility assessment (10 cases), artificial insemination (5 cases), sperm rescue (postmortem collection for cryopreservation, 5 cases), and sperm banking (in vivo collection for cryopreservation, 2 cases). Semen collection was successful (defined as at least 1 × 10^6^ spermatozoa) in 15 cases. The failures in obtaining spermatozoa were most probably due to (1) male infertility, (2) wrong age/non-breeding season, or (3) recent multiple copulations. The cases presented here confirm that although ART have been introduced into clinical practice, they are mostly used in cases of infertility, not as routine breeding tools. Higher involvement of zoological gardens and private breeders is required, as many chances for preservation of valuable material are lost.

## 1. Introduction

According to the IUCN red list, half of the wild felid species are, at present, classified as endangered or vulnerable [1]. Even those species, which have been classified globally as being of ‘least concern’, are protected at the national level (owing to the low number of individuals and existing threats: habitat loss, poaching, revenge killing, exotic pet trade, hybridization, etc.). One such example is the European wildcat, considered endangered in the Red Data Book of Poland [2]. To ensure the sustainability of populations, especially those highly endangered species, huge in situ and ex situ conservation efforts are being carried out throughout the world.

One of the methods of ex situ conservation is breeding endangered species in zoological gardens or specialized conservation centers. However, breeding in captivity is not always successful, due to, e.g., intraspecies aggression, incompatibility (rejection of a mating partner), inadequate sexual behavior, lack of libido, or incompetent mating [3]. This can be a serious problem in breeding canters, where an ‘ineffective’ individual/breeding pair cannot be easily replaced. The small number of individuals available in captivity and the difficulties with the exchange of animals between institutions are not conducive to maintaining genetic diversity. The solution could be to use artificial reproductive techniques (ART), such as cryopreservation of gametes collected during the lifetime or postmortem, as well as the use of artificial insemination (AI), in vitro fertilization (IVF), and embryo transfer (ET).

The development of ART for the domestic cat in the 1970s [4,5,6] raised hope that those techniques could be applied eventually to wild felids. Indeed, in 1981, the birth of the first AI-derived puma (*Puma concolor*) was reported [7], followed by the delivery of the first in vitro produced tiger (*Panthera tigris*) cub [8]. Since then, several litters have been obtained in different species (reviewed by [9]), and it has been constantly pointed out by different authors that methods and protocols developed on the domestic cats model could (and should) be used in programs designed to safeguard endangered feline species [10,11,12,13,14,15,16]. Recently, the birth of the world’s first IVF–ET-derived cheetah (*Acinonyx jubatus*) cubs was reported [17], showing that continuous progress is being made in this field and that the idea of introduction of ART in wild felids is still valid today.

In parallel to all the efforts focused on obtaining progeny via AI or ET, techniques for cryopreservation of gametes, reproductive tissues, and somatic cells have been developed in both the domestic cat and wild felids [14,15]. Cryobanking appears to be a crucial tool in maintaining biodiversity; stored material represents a ‘life insurance’ in case other conservation efforts fail. Also, it is easier to exchange cryopreserved material than live animal zoological gardens, and in this way, cryopreservation facilitates gene flow and reduction of homozygosity in the captive population. Apart from the major cryobanking initiatives (e.g., Smithsonian Institute, Frozen Ark), there are several reports on the creation of cryobanks dedicated to wild felids [18,19,20] or even to one species: the Iberian lynx (*Lynx pardinus*) [21].

Although these successes demonstrate that the application of ART for wild felids is possible, there is little information as to the extent to which those techniques are used in practice. To the authors’ knowledge, there is no organization gathering and publishing worldwide data in this field (similar to, e.g., International Embryo Technology Society reports for farm animals ET). Only a few publications describe collective data, such as those listing activities performed in neotropical felids [14,22] and reporting biobanking of feline material in Europe [18]. In our experience, many research groups perform ART in wild felids occasionally for nonscientific purposes (at zoo’s/owners’ requests), but those efforts are not reported, and the data obtained during such activities usually remain unpublished or are added to publications devoted to domestic cats [13]. Sharing of such data would contribute to: (1) increasing knowledge on gametes quality in captive wild felids; (2) providing information on how widely ART is incorporated into wild felid preservation; (3) encouraging other groups to become involved in this field.

Therefore, the aim of this manuscript is to present collective data on non-experimental semen collection in captive wild felids obtained by three European teams: from Wrocław University of Environmental and Life Sciences (Poland), Ghent University (Belgium), and Stepan Gzhytskyi National University of Veterinary Medicine and Biotechnologies Lviv (Ukraine).

## 2. Materials and Methods

### 2.1. Animals

In this report, we included data on semen collections performed in the years 2014–2019 in different species of wild felids kept in zoological gardens or privately owned in Poland, Ukraine, and Belgium. These include four cheetahs (*Acinonyx jubatus*), three servals (*Leptailurus serval*), two caracals (*Caracal caracal*), two ocelots (*Leopardus pardalis*), two sand cats (*Felis margarita*), and one individual from each of the following species: European lynx (*Lynx lynx*), jaguar (*Panthera onca*), leopard (*Panthera pardus*), lion (*Panthera leo*), Pallas cat (*Otocolobus manul*), snow leopard (*Panthera uncia*), and tiger (*Panthera tigris*). All animals were kept in conditions appropriate to their species, with access to an outdoor enclosure, being fed according to species requirements and being under the care of veterinarians who did not report any welfare concerns. The animals intended for breeding were kept with the females constantly or only during heat. Except in cases of postmortem semen collection, the males were healthy and in good condition. Semen collections were performed as a veterinary service; therefore, Ethic Committee approval was not required.

For postmortem semen collection, testicles were usually sent from the zoo to the laboratory within 12 h after the animal’s death was noted. In one case (European lynx), the testicles were collected 24 h after the animal’s death and arrived at the laboratory in 72 h after retrieval. The material was transported cooled and accompanied by a cover letter describing the case and the suspected reason for death.

### 2.2. Reagents

Most of the chemicals were obtained from Sigma-Aldrich (branch in a given country). Equex STM paste was purchased from Minitübe (branch or sales representative in a given country). The glycerol in Belgium was purchased from Novolab (Geraardsbergen, Belgium). The TRIS buffer, used as a basic semen extender, contained 3.02% (*w*/*v*) TRIS, 1.35% (*w*/*v*) citric acid, and 1.25% (*w*/*v*) fructose, in bidistilled water; pH was 6.5.

### 2.3. Anesthesia

All collections in live animals were performed under general anesthesia. Animals did not have access to food 24 h before anesthesia. The animals were sedated with medetomidine hydrochloride at 100–150 µg/kg of body weight, and im (Sedator^®^ 1.0 mg/mL, Novartis, Poland, or Domitor^®^, Orion Pharma, Belgium) combined with ketamine at 2.5–3.0 mg/kg, im (Bioketan^®^, Vetoquinol Polska, Poland or Ketamine^®^, Randlab, Belgium). In some cases, xylazine (Sedazin^®^ 20 mg/mL, Biowet Puławy, Poland) was used instead of medetomidine, or tiletamine with zolazepam (Zoletil^®^, Virbac, France) was used instead of ketamine. In some cases, additional pharmacological agents were administered intramuscularly: butorphanol, 0.3 mg/kg (Butomidor^®^ 5 mg/mL, Orion Pharma, Poland); atropine (Atropinum sulfuricum WZF, 0.5 mg/mL, Polfa Warszawa, Poland); midazolam (Midanium^®^ 5 mg/mL, Polfa Warszawa, Poland). The exact anesthetic protocols are provided as Supplementary Material S1.

Anesthesia was reversed after semen collection (30–60 min after the beginning of sedation) with an intramuscular injection of 200–312.5 μg/kg of atipamezole (Revetor^®^, Scanvet, Poland).

During semen collection, anesthesia and animal health were monitored by a separate designated person. Recovery after anesthesia was monitored by a zoo veterinarian.

### 2.4. Semen Collection

Semen collections were performed using different methods described below, at the request of the zoo/owners. The reasons for semen collection were: fertility check on suspicion of infertility (in case of no pregnancy, despite proper mating being observed), artificial insemination (when breeding failure was suspected to be caused by improper mating or when the female rejected a given male), or sperm recovery in vivo or postmortem for cryopreservation and gene banking.

All semen collections in live animals were performed in the zoo/animal homes, except for one serval, which was brought to the veterinary clinic for magnetic resonance examination. Different methods were used in different cases; in some animals, electroejaculation was performed after unsuccessful collection by urethral catheterization.

#### 2.4.1. Postmortem Recovery of Epididymal Spermatozoa

Spermatozoa were collected immediately after receiving the testicles with epididymides. The caudae epididymides were dissected from the testes and cleaned of connective tissues and visible blood vessels. The caudae epididymides were then placed in 1 mL of prewarmed TRIS buffer in a glass Petri dish and minced using a scalpel blade. After a 10-min incubation, epididymal tissues were removed and a suspension of spermatozoa was filtered (CellTrics 30 µm, Partec) into an Eppendorf tube.

#### 2.4.2. Urethral Catheterization

Approximately 10–20 min after injection of medetomidine, urethral semen was collected by introducing an open-end urinary catheter into the urethra. In small felids (sand cats, servals, and ocelots), a 1.0 mm × 130 mm tomcat urinary catheter was used. For larger felids, a dog urinary catheter (2.0 mm × 300 mm or 2.6 mm × 500 mm) was used. Two exceptions were the jaguar, for which a 1.0 tomcat urinary catheter was also used, as previously described [23], and a caracal, for which a 2.0 dog catheter was used. To avoid entering the urinary bladder, the length from the tip of the penis to the cranial symphysis pelvis was measured and marked on the catheter, indicating the estimated length of catheter insertion. In most cases, the catheter was inserted repetitively (2–4 times), each time a bit deeper, to collect all the spermatozoa, but to avoid entering the urinary bladder. After several seconds, when sperm cells entered the catheter by capillary forces, the catheter was removed from the urethra and the sperm sample was placed in a sterile Eppendorf tube containing 200 µL of prewarmed TRIS buffer. An exception was a caracal whose spermatozoa were suspended directly in freezing extender A (see below).

#### 2.4.3. Electroejaculation

In several cases, when urethral semen collection was unsuccessful, electroejaculation was performed. The rectum was emptied of feces and a lubricated probe was inserted into the rectum. The size of the probe was matched to the size of each species. The probe was placed with the electrodes directed ventrally on top of the male reproductive glands. Then, a few series of increasing voltage electric stimuli (ranging from 2–6 volts) were administered, using a controlled voltage electrostimulator. The electrostimulation protocols (number of stimuli and their organization into a series) followed data from the literature [24,25,26,27]. Semen was collected in an empty, sterile, warm 1.5 mL Eppendorf tube or 15 mL Falcon conical tube (tiger).

### 2.5. Semen Evaluation

Immediately after semen collection, the volume of the sample was measured using an automatic pipette. Motility was subjectively estimated by two researchers using a phase contrast microscope with a warming plate (37 °C) at ×40 magnification. The concentration of the sample was determined using a Bürker or Thoma chamber. The percentages of morphologically normal and abnormal spermatozoa (possessing at least one of the abnormalities: abnormal head size or shape, detached head, midpiece defects, bent or coiled tail, or proximal or distal droplet), as well as vitality, were evaluated on a phase contrast microscope (×100 objective with immersion oil) after eosin–nigrosin staining. For both vitality and morphology, 100 or 200 spermatozoa were counted and classified. When only a few spermatozoa were obtained, concentration, vitality, and morphology were not assessed.

### 2.6. Sperm Freezing and Thawing

Sperm cryopreservation was performed immediately after assessment using two different protocols: Protocol 1 according to Niżański et al. [28] and Protocol 2 according to Axner et al. [29].

#### 2.6.1. Protocol 1

After centrifugation at 620× *g* for 5 min, the supernatant was aspirated and discarded. The sperm pellet was re-suspended at room temperature in a freezing extender containing TRIS buffer supplemented with 20% egg yolk (*v*/*v*), 6% glycerol (*v*/*v*), 1% Equex paste (*v*/*v*) and 0.5% gentamicin (*w*/*v*). The final concentration of spermatozoa was adjusted to 40 × 10^6^ spermatozoa per mL. After resuspension, sperm samples were cooled to the temperature of 5 °C within 1 h, equilibrated at 5 °C for 1.5 h, loaded into pre-cooled 0.25 mL straws, and placed 5 cm above the surface of liquid nitrogen for 10 min. The straws were then plunged into liquid nitrogen and kept at −196 °C until further evaluation. Thawing was carried out by immersion of a straw in a 37 °C water bath for 30 s, and the contents were emptied into an Eppendorf tube.

#### 2.6.2. Protocol 2

The semen specimen was ejected from the catheter into an Eppendorf tube which contained 200 μL of freezing extender A (TRIS buffer supplemented with 20% (*v*/*v*) egg yolk, 3% glycerol (*v*/*v*) and 0.5% gentamicin (*w*/*v*)). After taking samples for sperm analysis, the Eppendorf tube was gradually chilled to 4 °C. After 2 h, 200 μL of chilled (4 °C) freezing extender B (TRIS buffer supplemented with 20% (*v*/*v*) egg yolk, 6% glycerol (*v*/*v*), 1% Equex paste (*v*/*v*), and 0.5% gentamicin (*w*/*v*)) was added. The semen was loaded into 0.25 mL straws, but the straws were only half filled, sealed, and placed in liquid nitrogen vapor, 4 cm above the liquid nitrogen surface. After 20 min, the straws were plunged into liquid nitrogen. Thawing was carried out by placing the straw in a 37 °C water bath for 30 s, and the straw contents were then emptied into a 1.5 mL Eppendorf tube which already contained 125 μL of prewarmed (37 °C) thawing medium (TRIS buffer with 0.5% gentamicin).

Since collected and cryopreserved spermatozoa were considered to be valuable samples, thawing to evaluate post-thaw quality was carried out only in two cases.

## 3. Results

**Figure 1:**
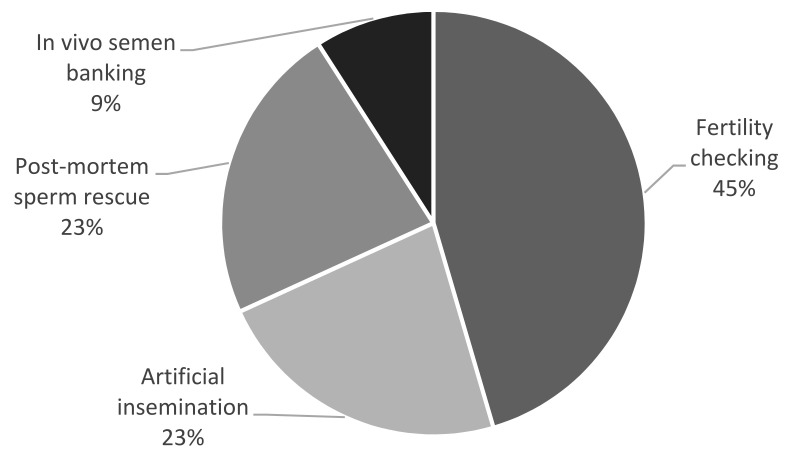
Reasons for semen collection in the presented cases (*n* = 22).

Semen was collected on 19 occasions from 20 individuals, resulting in a total of 22 semen collection attempts (in two males, semen was collected twice over an interval of a few months, while in three cases, two animals were examined on the same occasion). The most common reason for semen collection was infertility—either for diagnostic purposes (checking fertility in the case of no pregnancy, despite proper mating; 10/22 cases) or as a treatment for artificial insemination (when breeding failure was suspected to be caused by improper mating or when the female rejected a given male; 5/22 cases). There were five attempts to recover sperm postmortem from the epididymis, and semen was collected in vivo for cryobanking only in two cases (Figure 1).

The demographic data of the males included in this study, and their basic semen quality, are summarized in Table 1, with the details of each case provided as Supplementary Material S2. In total, semen collection was successful (defined as recovering at least 1 × 10^6^ spermatozoa) in 15 cases. Urethral catheterization allowed the collection of a high number of sperm cells (30–236 × 10^6^) in five cases, while in the remaining cases, either a low number of sperm (0.2–8.5 × 10^6^, 4 samples) or no spermatozoa were obtained (4 samples). In two cases, the use of electroejaculation after unsuccessful collection of urethral semen allowed the collection a high number of sperm cells (tiger—120 × 10^6^, leopard—25.2 × 10^6^), while in the remaining cases, no semen was obtained. A similar number of sperm was obtained from a male cheetah by both methods (30.5 × 10^6^ and 25.6 × 10^6^). When no spermatozoa were collected by either method, the zoo/owners were unwilling to collect semen again after few days/weeks and such a male was considered to be infertile. In two cases where a low number of sperm cells were obtained (cheetah: 6 × 10^6^; sand cat: 8.5 × 10^6^), these males proved to be fertile at the time of semen collection (cheetah) or one year later (sand cat). Only a few sperm cells were collected from two servals which had previously sired multiple offspring and were active breeders at the time of collection.

Of the five attempts at postmortem epididymal sperm collection, two were successful (ocelot: 1228.5 × 10^6^ total sperm count; lion: 95 × 10^6^ total sperm count). The failure in the other cases was most probably due to testicular degeneration (caracal) or collection outside the breeding season (European lynx and Pallas cat). The quality of the sperm obtained varied greatly (motility ranged from 95% to 5%) and was worse in the cases of postmortem collection (<40% motility). Teratozoospermia was observed in almost all individuals (range of normal sperm cells was 10% to 55%), and was the most severe in cheetahs.

Cryopreserved semen was thawed for assessment in two cases. In the caracal (individual 2, semen collected in vivo for cryopreservation with protocol 2), 50% of the spermatozoa were motile post-thaw, with 88% live, and 20% morphologically normal. In the ocelot (individual 1, spermatozoa collected postmortem and cryopreserved with protocol 1), post-thaw motility, vitality, and normal morphology were 15%, 32%, and 32.5%, respectively.

The details about artificial inseminations are provided in Table 2. None resulted in the birth of offspring, probably due to reproductive abnormalities in the female (three cases). One procedure (serval) was abandoned due to an insufficient number of spermatozoa in the insemination dose.

## 4. Discussion

The cases presented here demonstrate that ART are used clinically in captive wild felids that are in zoos and privately owned, even though on a small scale. Analysis of the reasons for semen collection showed that the main motive for animal owners to search for ART is related to cases of unsuccessful mating. In terms of semen banking, more cases made use of postmortem semen collection and only two attempts at in vivo collection were made. This suggests that ART in wild felids is still considered to be more of an emergency approach than a routine procedure. There may be several reasons for this situation.

According to the authors’ personal observations (data not reported), one reason that ART are not widely used in wild animals could be due to a fear of anesthesia; animal owners are unwilling to anesthetize animals in absence of veterinary indications, and still have significant concerns when it is required. However, it is not yet possible to collect semen from wild animals that have not been sedated. As each instance of anesthesia, even under ideal circumstances, carries some risk of animal death [33], the fear of losing a valuable individual is justified. Fortunately, more and more veterinarians are becoming skilled in chemical immobilization of particular species, which helps to lower the risk of peri-anesthetic mortality and may contribute to more frequent use of ART in captive wild felids.

If fear of ‘unnecessary’ anesthesia is the reason that hampers the use of ART, then one of the solutions would be the collection of semen when sedation is required for other veterinary procedures. Among our cases, only one was performed ‘on occasion’, showing that there is a space for an increase in this approach. Zoological gardens should be encouraged to contact researchers if the sedation of an animal is planned, so that the opportunity to collect highly valuable reproductive material is not lost.

Another reason could be ethical concerns related to the use of electroejaculation, especially in Europe, where this method is banned for use in livestock in several countries [34]. For years, electroejaculation was the only option for the collection of semen in wild felids, but since 2008, there has been a novel method of semen collection in felids: urethral catheterization after medetomidine administration [35]. The use of this method has been reported so far in only a few wild cat species: lions [36], Asiatic golden cats [37], jungle cats [38], tigers [36,39], cheetahs [36,39], black panthers [39], cougars [39], and jaguars [23]. This report extends this list with the inclusion of caracal, ocelot, and sand cat.

However, it should be highlighted that in some cases where urethral catheterization was unsuccessful, electroejaculation allowed the collection of semen (tiger, leopard), similar to what has been reported for servals and lynxes [40], but contrary to studies on the domestic cat [35]. This may suggest that urethral catheterization requires further adaptation for a given species (e.g., medetomidine dosage, catheter size, and depth of catheter insertion). A good example of a species-specific approach is the collection of urethral semen in jaguars, in which a relatively small domestic cat urinary catheter was used successfully [23], which was confirmed in the present study.

Furthermore, several males described in this study (cheetah, servals) were fertile before semen collection, but no or little spermatozoa were obtained from these males by urethral catheterization or electroejaculation. It is well known from human studies [41] as well as from our observations in domestic cats, that semen quality may show fluctuations and differ significantly between consecutive collections. Additionally, studies in the domestic cat showed that on average, five consecutive ejaculations lead to a decrease in the total number of spermatozoa to below 5 × 10^6^, and a rest period (abstinence) of around 6 days was necessary to recover [42]. Considering mating patterns in felids (multiple copulations during the estrus period) and that these males were active reproducers, the failure in semen collection was probably caused by ‘reproductive exhaustion’ of the male. This is the rationale for the rule that for fertility assessment, semen should be collected after at least 6 days of abstinence and in the case of bad semen quality, and it should be examined more than once (preferably three times).

Finally, a low success rate may be a discouraging factor. Although achievements in the use of ART in wild felids were reported and kittens were born after AI or ET [7,8,9,17], they can be considered as isolated achievements. Despite years of studies, the pregnancy rate after AI or ET is still low in felids, with a live birth rate ranging from 9% to 40% in different species (reviewed by [9]). The key points in evaluating the success of AI in felids are: (1) hormonal stimulation of females, (2) timing of insemination, (3) sperm quality, and (4) technique and location of sperm deposition [16]. A hormonal stimulation protocol utilizing eCG and porcine LH (pLH) together with laparoscopic oviductal artificial insemination resulted in high pregnancy rates in the domestic cat and was successful in various wild felids [43]. The use of pLH is superior to hCG, as it does not induce postovulatory ovarian stimulation [43]; however, pLH is not available in Europe. Deposition of spermatozoa in the oviduct allows a marked reduction in the number of sperm cells required (to 1–10 × 10^6^ motile spermatozoa), which is of great importance in felids, which generally produce semen of poor quality [43]. On the other hand, the oviductal laparoscopic technique requires greater technical skills and is considered to be more invasive than endoscopic transcervical intrauterine insemination used in this study. Another approach, transcervical intrauterine insemination in natural estrus and stimulation of ovulation by GnRH, has also been reported to be successful [37,44]. The disadvantages of this protocol are: poorer time flexibility (natural estrus) and the requirement for a high number of spermatozoa (100 × 10^6^ motile spermatozoa recommended for lions [44]). It is impossible to state the reason for failure in the cases described in this study, but a history of previous unsuccessful matings and reproductive abnormalities observed in three females could have been the source of problems in these cases. Therefore, we stipulate that AI should only be performed in healthy animals, to increase the success rate and to convince owners that it can be a valuable tool for breeding programs and the exchange of genetic material. At the same time, more studies are required to establish optimal procedures and protocols for a given species. More work and attention should also be devoted to oocyte retrieval, in vitro fertilization, and embryotransfer, as methods which allow the preservation and use of female gametes, and the use of bad quality/low number of spermatozoa (>10 × 10^6^ or >1.5 × 10^6^ motile sperm required for AI or IVF, respectively, [18]). However, the use of these techniques is limited by the requirement for a specialized in vitro fertilization laboratory.

The success rate was also low for postmortem sperm rescue; in this report, we managed to obtain a large amount of semen suitable for cryopreservation in only two out of five individuals (40%), and other authors reported similar results [18,21]. In most cases, the problem is that spermatozoa are collected from very old or diseased animals, which already had diminished semen quality [18], or conversely, from young, prepubertal males [21]. For species showing seasonality, sperm recovery outside of the breeding season is also unsuccessful [21]. Taking all this into account, it is crucial to collect and preserve material from genetically valuable individuals in vivo, when they are still healthy and in the reproductive phase.

Another reason for poor epididymal semen quality may be the time factor (from animal death to delivery to the andrology laboratory). It is possible to collect viable, motile domestic cat spermatozoa within up to 72 h of storage at 4 °C [45,46], although motility decreases dramatically with time [45]. The best approach would be sperm retrieval within 24 h, both in the domestic cat [45,46] and in wild felids [18]. Therefore, a good notification and logistics system should be developed to facilitate transportation of the material in the shortest time possible. Also, the large number of centers spread throughout Europe, where material could be sent for gametes retrievals and banking, would allow a higher degree of preservation of genetic resources in the case of animal death.

## 5. Conclusions

The cases presented here show that there is an interest in semen collection from captive wild felids for veterinary reasons (fertility check and treatment of infertility). Semen assessment is an indispensable element of the andrological examination of the male and here, it helped in the diagnosis of infertility. Artificial insemination was not successful in the treatment of infertility, which we attribute to female problems and the overall low success rate of this technique in felids. Failure in sperm retrieval postmortem and the lower semen quality from these collections highlight the importance of banking material from live animals. However, the only two cases of in vivo semen collection for cryopreservation reported here suggest that the use of ART for protection of genetic diversity is still underdeveloped. There is a need for a wider involvement of zoos, especially in terms of sending the material in case of animal death, and collection of spermatozoa during scheduled anesthesia.

Semen collection from wild felids, usually performed in semi-field conditions, can be a challenge for the veterinary team, and stressful for the animals, their owners, and the researchers. However, this should not be a reason to limit the use of ART in endangered species. Wider training of researchers and vets will ensure that the procedure will be safe and successful, and the existence of a higher number of skilled teams will contribute to better access to these services. Also, there should be international cooperation of all teams to support the exchange of knowledge and experience and to hasten progress in this field.

## Figures and Tables

**Table 1:** Semen parameters in wild felids (*n* = 22).

Species	Individual	Age [Years]	Purpose of Semen Collection	Method of Collection	Total Sperm Count [×10^6^]	Motility [%]	Viability [%]	Normal Morphology [%]
Caracal (*Caracal caracal*)	1	No data	Postmortem sperm rescue	EP	1	5	63.5	31.5
	2	3	Cryopreservation	CT	23.6	80	93	37
Cheetah (*Acinonyx jubatus)*	1	5	Fertility check	CT	6	30	79	8
				EE [24]	No spermatozoa	-	-	-
	2	5	Fertility check	CT	30.5	50	78	7.5
				EE [24]	25.6	80	70	10
	3	10	Fertility check	CT	12	3 progressive	-	-
	4	10	Fertility check	CT	57.5	80 (50 in one sample)	-	-
European lynx (*Lynx lynx)*	1	1.5	Postmortem sperm rescue	EP	No spermatozoa	-	-	-
Jaguar (*Panthera onca*)	1	6	AI	CT	98	75	73	49
Leopard *(Panthera pardus)*	1	5	AI	CT	No spermatozoa	-	-	-
				EE [24]	25.2	60	62.5	-
Lion (*Panthera leo)*	1	16	Postmortem sperm rescue	EP	95	30	74	32
Ocelot (*Leopardus pardalis*)	1	10	Postmortem sperm rescue	EP	1 228.5	40	63	33.5
	2	9	Fertility check	CT	95.5	95	-	-
			AI	CT	99.2	95	96.5	-
Pallas Cat (*Otocolobus manul*)	1	9	Postmortem sperm rescue	EP	Few immotile sperm cells	0	-	-
Sand cat (*Felis margarita)*	1	12	Fertility check	CT	8.5	50	81.5	20.5
				EE [27]	1.8	80	64.5	35
	2	12	Fertility check	CT + EE [27]	No spermatozoa	-	-	-
Serval *(Leptailurus serval)*	1	12	Cryopreservation	CT	Few sperm cells	5	-	-
				EE [26]	No spermatozoa	-	-	-
	2	7	AI	CT	0.2	40	78	-
				EE [26]	No spermatozoa	-	-	-
	3	5	Fertility check	CT + EE [26]	No spermatozoa	-	-	-
Snow leopard (*Panthera uncia*)	1	10	Fertility check	CT	No spermatozoa	-	-	-
				EE [25]	Few immotile sperm cells	0	-	-
Tiger (*Panthera tigris*)	1	4	Fertility check	CT	No spermatozoa	-	-	-
				EE [24]	120	65	-	46
		5	AI	EE [24]	100	60		

AI—artificial insemination; CT—urethral catheterization; EP—epididymal slicing; EE—electroejaculation. Numbers in brackets [24,25,26,27] indicate references for electroejaculation protocol.

**Table 2:** Artificial inseminations (AI) in wild felids. In all cases, fresh spermatozoa (collected within one hour before procedure) were deposited in the uterus by transcervical intrauterine endoscopic insemination.

Species	Female Age [Years]	Hormonal Stimulation Protocol	Motile Spermatozoa Inseminated [×10^6^]	Pregnancy Outcome	Notes
Jaguar (*Panthera onca*)	7	300 IU eCG followed by 225 IU hCG 80 h later. AI 45 h after hCG [30].	73.5	Not pregnant	During insemination, a large amount of thick mucous discharge was revealed in the vagina.
Leopard *(Panthera pardus)*	11	300 IU eCG followed by 225 IU hCG 80 h later. AI 45 h after hCG	15	Not pregnant	During insemination bloody discharge was revealed in the vagina.
Ocelot (*Leopardus pardalis*)	9	500 IU eCG followed by 250 IU hCG 80 h later. AI 40 h after hCG injection [31].	94	Not pregnant	The female was diagnosed and treated for pyometra three months before AI.
Serval (*Leptailurus serval*)	4	-	-	-	AI abandoned due to the insufficient number of spermatozoa (0.2 × 10^6^)
Tiger (*Panthera tigris*)	6	1000 IU eCG, followed by 750 IU hCG 80 h later. AI 45 h after hCG. [32]	60	Not pregnant	The zookeepers reported behavioral changes and abdominal distention that could have been signs of gestation, but confirmation of pregnancy was not possible.

## Data Availability

Not applicable—All data are presented in the manuscript.

## Supplementary Materials

The following supporting information can be downloaded at: https://www.mdpi.com/article/10.3390/ani12070871/s1. Table S1: Anesthetic protocols used in the study. Material S2: Description of cases.

## References

[1] The IUCNRed List of Threatened Species Version 2021-3. https://www.iucnredlist.org.

[2] Wolsan M., Okarma H., Głowaciń Z. (2001). Felis silvestris Schreber, 1775. Żbik. Polish Red Data Book of Animals.

[3] Von Schmalz-Peixoto K.E. (2003). Factors Affecting Breeding in Captive Carnivora. Ph.D. Thesis.

[4] Sojka N.J., Jennings L.L., Hamner C.E. (1970). Artificial insemination in the cat (*Felis catus* L.). Lab. Anim. Care.

[5] Platz C.C., Wildt D.E., Seager S.W. (1978). Pregnancy in the domestic cat after artificial insemination with previously frozen spermatozoa. J. Reprod. Fertil..

[6] Hamner C.E., Jennings L.L., Sojka N.J. (1970). Cat (*Felis catus* L.) spermatozoa require capacitation. J. Reprod. Fertil..

[7] Moore H.D., Bonney R.C., Jones D.M. (1981). Successful induced ovulation and artificial insemination in the puma (*Felis concolor*). Vet. Rec..

[8] Donoghue A.M., Wolf P., Gross T., Armstrong L., Tilson R.L., Wildt D.E. (1990). In vitro fertilization and embryo development in vitro and in vivo in the tiger (*Panthera tigris*). Biol. Reprod..

[9] Veraguas D., Echeverry D., Castro F., Rodriguez-Alvarez L. (2017). Applied Biotechnologies in the Conservation of Wild Felids: In Vitro Embryo Production and Cellular Regenerative Therapies. Big Cats.

[10] Wildt D.E., Schiewe M.C., Schmidt P.M., Goodrowe K.L., Howard J., Phillips L.G., O’brien S.J., Bush M. (1986). Developing animal model systems for embryo technologies in rare and endangered wildlife. Theriogenology.

[11] Pope C.E. (2000). Embryo technology in conservation efforts for endangered felids. Theriogenology.

[12] Swanson W.F. (2006). Application of assisted reproduction for population management in felids: The potential and reality for conservation of small cats. Theriogenology.

[13] Cocchia N., Ciani F., El-Rass R., Russo M., Borzacchiello G., Esposito V., Montagnaro S., Avallone L., Tortora G., Lorizio R. (2010). Cryopreservation of feline epididymal spermatozoa from dead and alive animals and its use in assisted reproduction. Zygote.

[14] Silva H.V.R., Silva A.R., Da Silvada L.D.M., Comizzoli P. (2019). Semen Cryopreservation and Banking for the Conservation of Neotropical Carnivores. Biopreserv. Biobank..

[15] Jewgenow K., Zahmel J. (2020). Preservation of female genetic resources in feline species. Theriogenology.

[16] Thongphakdee A., Sukparangsi W., Comizzoli P., Chatdarong K. (2020). Reproductive biology and biotechnologies in wild felids. Theriogenology.

[17] Crosier A.E., Lamy J., Bapodra P., Rapp S., Maly M., Junge R., Haefele H., Ahistus J., Santiestevan J., Comizzoli P. (2020). First Birth of Cheetah Cubs from In Vitro Fertilization and Embryo Transfer. Animals.

[18] Fernandez-Gonzalez L., Jewgenow K., Müller K., Zahmel J. (2019). Felid-Gamete-Rescue Within EAZA—Efforts and Results in Biobanking Felid Oocytes and Sperm. JZAR.

[19] Amstislavsky S.Y., Kozhevnikova V.V., Muzika V.V., Kizilova E.A. (2017). Reproductive Biology and a Genome Resource Bank of Felidae. Ontogenez.

[20] Kochan J., Niżański W., Moreira N., Cubas Z.S., Nowak A., Prochowska S., Partyka A., Młodawska W., Skotnicki J. (2019). ARTs in Wild Felid Conservation Programmes in Poland and in the World. J. Vet. Res..

[21] Leon-Quinto T., Simon M.A., Cadenas R., Jones J., Martinez-Hernandez F.J., Moreno J.M., Vargas A., Martinez F., Soria B. (2009). Developing biological resource banks as a supporting tool for wildlife reproduction and conservation The Iberian lynx bank as a model for other endangered species. Anim. Reprod. Sci..

[22] Swanson W.F., Brown J.L. (2004). International training programs in reproductive sciences for conservation of Latin American felids. Anim. Reprod. Sci..

[23] Araujo G.R., Paula T.A.R., Deco-Souza T., Morato R.G., Bergo L.C.F., Silva L.C.D., Costa D.S., Braud C. (2018). Comparison of semen samples collected from wild and captive jaguars (*Panthera onca*) by urethral catheterization after pharmacological induction. Anim. Reprod. Sci..

[24] Wildt D.E., Phillips L.G., Simmons L.G., Chakraborty P.K., Brown J.L., Howard J.G., Teare A., Bush M. (1988). A comparative analysis of ejaculate and hormonal characteristics of the captive male cheetah, tiger, leopard, and puma. Biol. Reprod..

[25] Johnston L.A., Armstrong D.L., Brown J.L. (1994). Seasonal effects on seminal and endocrine traits in the captive snow leopard (*Panthera uncia*). J. Reprod. Fertil..

[26] Pukazhenthi B., Spindler R., Wildt D., Bush L.M., Howard J. (2002). Osmotic properties of spermatozoa from felids producing different proportions of pleiomorphisms: Influence of adding and removing cryoprotectant. Cryobiology.

[27] Herrick J.R., Bond J.B., Campbell M., Levens G., Moore T., Benson K., D’Agostino J., West G., Okeson D.M., Coke R. (2010). Fecal endocrine profiles and ejaculate traits in black-footed cats (*Felis nigripes*) and sand cats (*Felis margarita*). Gen. Comp. Endocrinol..

[28] Niżański W., Dejneka J., Klimowicz M., Dubiel A. (2005). Ocena wybranych właściwości plemników najądrzowych kota domowego i ich konserwacja w niskich temperaturach. Med. Weter.

[29] Axnér E., Hermansson U., Linde-Forsberg C. (2004). The effect of Equex STM paste and sperm morphology on post-thaw survival of cat epididymal spermatozoa. Anim. Reprod. Sci..

[30] Jimenez Gonzalez S., Howard J.G., Brown J., Grajales H., Pinzón J., Monsalve H., Moreno M.A., Jimenez Escobar C. (2017). Reproductive analysis of male and female captive jaguars (*Panthera onca*) in a Colombian zoological park. Theriogenology.

[31] Da Paz R.C., Dias E.A., Adania C.H., Barnabe V.H., Barnabe R.C. (2006). Ovarian response to repeated administration of alternating exogenous gonadotropin regimens in the ocelot (*Leopardus pardalis*) and tigrinus (*Leopardus tigrinus*). Theriogenology.

[32] Donoghue A.M., Byers A.P., Johnston L.A., Armstrong D.L., Wildt D.E. (1996). Timing of ovulation after gonadotrophin induction and its importance to successful intrauterine insemination in the tiger (*Panthera tigris*). J. Reprod. Fertil..

[33] Arnemo J.M., Ahlqvist P., Andersen R., Berntsen F., Ericsson G., Odden J., Brunberg S., Segerström P., Swenson J.E. (2006). Risk of capture-related mortality in large free-ranging mammals: Experiences from Scandinavia. Wildl. Biol..

[34] Palmer C.W. (2005). Welfare aspects of theriogenology: Investigating alternatives to electroejaculation of bulls. Theriogenology.

[35] Zambelli D., Prati F., Cunto M., Iacono E., Merlo B. (2008). Quality and in vitro fertilizing ability of cryopreserved cat spermatozoa obtained by urethral catheterization after medetomidine administration. Theriogenology.

[36] Lueders I., Luther I., Scheepers G., Van der Horst G. (2012). Improved semen collection method for wild felids: Urethral catheterization yields high sperm quality in African lions (*Panthera leo*). Theriogenology.

[37] Lueders I., Ludwig C., Schroeder M., Mueller K., Zahmel J., Dehnhard M. (2014). Successful nonsurgical artificial insemination and hormonal monitoring in an Asiatic golden cat (*Catopuma temmincki*). J. Zoo Wildl. Med..

[38] Kheirkhah M.S., Sisakht M.M., Mohammadsadegh M., Moslemi H.R. (2017). Sperm evaluation of Jungle Cat (*Felis chaus*) obtained by urethral catheterization (CT) after medetomidine administration. Theriogenology.

[39] Cunto M., Küster D.G., Bini C., Cartolano C., Pietra M., Zambelli D. (2015). Influence of Different Protocols of Urethral Catheterization after Pharmacological Induction (Ur.Ca.P.I.) on Semen Quality in the Domestic Cat. Reprod. Domest. Anim..

[40] Gonzalez R., Moresco A., Miller A., Bateman H., Vansandt L., Dembiec D., Ista A., Swanson W. (2019). 101 Assessment of semen traits in servals (*Leptailurus serval*) and Canada lynx (*Lynx canadensis*). Reprod. Fertil. Dev..

[41] Mallidis C., Howard E.J., Baker H.W. (1991). Variation of semen quality in normal men. Int. J. Androl..

[42] Tsutsui T., Oba H., Fujimoto S., Toyonaga M. (2012). Spermatogenic function in cats. Reprod. Domest. Anim..

[43] Swanson W.F. (2018). Practical application of laparoscopic oviductal artificial insemination for the propagation of domestic cats and wild felids. Reprod. Fertil. Dev..

[44] Callealta I., Ganswindt A., Malan M., Lueders I. (2019). Non-surgical artificial insemination using a GnRH analogue for ovulation induction during natural oestrus in African lions (*Panthera leo*). Theriogenology.

[45] Toyonaga M., Sato Y., Morita M., Watanabe M., Oba H., Mizutani T., Hori T., Tsutsui T. (2010). The qualities of cryopreserved epididymal sperm collected from feline epididymides stored at low temperature. J. Vet. Med. Sci..

[46] Gañán N., Gomendio M., Roldan E.R. (2009). Effect of storage of domestic cat (*Felis catus*) epididymides at 5 degrees C on sperm quality and cryopreservation. Theriogenology.

