# Supplementary material for: How Can We Introduce ART into Wild Felid Conservation in Practice? Joint Experience in Semen Collection from Captive Wild Felids in Europe
Source: Animals (Basel). 2022 Mar 30;12(7):871. doi: 10.3390/ani12070871 (PMC8997001; doi:10.3390/ani12070871)
Supplement: Supplementary file 1 [file animals-12-00871-s001.zip › Suppl Material S2.pdf]

Supplementary material S2: description of cases included in the manuscript

Case 1: postmortem semen collection in a caracal

A caracal died in a zoo, no data on age and previous fertility were provided. Testicles were collected immediately after noticing the death of the animal and arrived at the laboratory in 12 hours. There was an apparent asymmetry of the testicles (the weight of the right testicle was 389 mg, while the weight of the left testicle was 910 mg). The slicing of the caudae epididymides resulted in collection of $1x10^6$ spermatozoa (mot. 5%) only from the left epididymis, no spermatozoa were found in the right one. The testicles were sent for histopathological examination, which revealed testicular degeneration.

Case 2: semen collection for cryopreservation in a caracal

A privately owned three-year-old male caracal, the owner of which requested semen cryopreservation. The male had fathered one litter, the two kittens were three weeks old at the time of semen collection. Semen was successfully collected by urethral catheterization. In total, four catheterizations, 100 μL of semen were collected, containing $23.6x10^6$ sperm cells. Immediately after collection, sperm motility was estimated to be 80%. A high percentage of 93% live sperm was observed, but only 37% of the spermatozoa showed normal morphology. In total, four straws of 125 μL were cryopreserved according to protocol 2. After thawing a straw, approximately 50% of the spermatozoa were motile, the percentage of live sperm was 88% and 20% of the spermatozoa was morphologically normal.

Case 3: fertility assessment in two cheetahs

Two five-year-old cheetahs (full brothers) were kept in the zoo together with a seven-year-old female. The female copulated multiple times only with one of them (dominant), rejecting the other (described as timid), but no offspring was obtained. The zoo asked to check the quality of semen in those cheetahs. Semen was collected from both males by urethral catheterization, followed by electroejaculation. The first male (the one chosen by the female) had a low number of spermatozoa ($6x10^6$ urethral, no sperm after electroejaculation) of poor motility (30%). From the second male (rejected by the female) $30.5x10^6$ of urethral spermatozoa (50% motility) and $25.6x10^6$ of electroejaculated spermatozoa (80% motility) were collected. Spermatozoa of both males showed similarly bad sperm morphology (<10% of normal spermatozoa). The diagnosis was made that probably the first male is infertile/subfertile, and the zoo staff was asked to change the breeding management of these cheetahs (to isolate the first male from the female). If the female would still reject the second male, artificial insemination was planned. However, shortly after the female appeared to be pregnant and two cubs were born 87 days after semen check-up. The father was the first male. As the duration of gestation in cheetahs is 94.2 ± 0.5 days, fertilization must have taken place just before semen collection. We hypothesize that the failure to obtain semen from the first male was caused by multiple mating prior to the visit to the zoo.

The zoo agreed to donate the second cheetah's semen to the Wrocław Semen Bank of Companion and Wild Animals – four straws ($10x10^6$ sperm each) were cryopreserved immediately after collection according to Protocol 1 and are now stored in the Bank.

Case 4: fertility assessment in the other two cheetahs

The two ten-year-old cheetahs were housed in the same zoo. Both mated multiple times with a five-year-old female, but no offspring were obtained. The zoo asked for fertility checks in those cheetahs. Semen was collected from both males by urethral catheterization. The first male gave $12x10^6$ spermatozoa with very poor motility (3% progressive motility). The second male was catheterized four times, giving in total $57.5x10^6$ of urethral spermatozoa (50% motility in the second sample, 80% in the third and fourth). The female was examined by vaginal cytology, vaginal endoscopy, and ultrasound. Paraovarian cysts and cystic endometrial hyperplasia were diagnosed. The diagnosis was made that it was a female infertility.

Case 5: postmortem semen collection in an European lynx

An 18-month-old European lynx suddenly died with respiratory symptoms in a zoo. On autopsy, lung congestion was found. The testicles were collected 24 hours after animal death and arrived at the laboratory in 72 hours. Spermatozoa were not found in the epididymis, probably due to young age or non-breeding season (November).

Case 6: semen collection for artificial insemination in a jaguar

A six-year-old jaguar, living in zoo, was kept together with a seven-year-old female. Copulation attempts were noticed, but no offspring were obtained. For the zoo keepers, the copulation seemed to be improper and the zoo asked for artificial insemination. The female was hormonally stimulated by injection of 300 IU of eCG to induce estrus and then, 80 hours later, 225 IU of hCG was administered to induce ovulation [31]. Endoscopic transcervical artificial insemination was scheduled 45 hours after hCG. Semen was collected by urethral catheterization and $98.5x10^6$ spermatozoa of good motility (75% motility) were obtained. All spermatozoa were deposited in the uterus. During insemination, a large amount of thick mucous discharge was revealed in the vagina. Confirmation of pregnancy was not possible and there was no delivery.

Case 7: semen collection for artificial insemination in a leopard

A five-year-old Persian leopard was kept together with an 11-year-old female in the zoo. Zoo keepers observed copulations, but no offspring was born. The zoo requested artificial insemination. The female was hormonally stimulated by injection of 300 IU of eCG to induce estrus and then, 80 hours later, 225 IU of hCG was administered to induce ovulation. Endoscopic transcervical artificial insemination was scheduled 45 hours after hCG. Semen collection by urethral catheterization was unsuccessful (no spermatozoa obtained), but electroejaculation allowed to collect $25.2x10^6$ spermatozoa (60% motility). All spermatozoa were deposited in the uterus by endoscopic transcervical insemination. During insemination

bloody discharge was revealed in the vagina. The female did not get pregnant.

Case 8: postmortem semen collection in a lion

A sixteen-year-old lion living in a zoo was euthanized due to serious health problems. Testicles were collected right after animal death and arrived at the laboratory in 12 hours. The slicing of the caudae epididymides resulted in the collection of 95×10^6 spermatozoa (30% motile, 74% live and 32% of normal morphology), which were cryopreserved according to protocol 1. Five 0.25mL straws (10×10^6 spermatozoa each) and five straws of 0.125mL (cut half, 5×10^6 each) were deposited in the Wrocław Semen Bank of Companion and Wild Animals.

Case 9: postmortem semen collection in an ocelot

An ocelot kept in the zoo died at the age of 10 due to purulent multiple organs inflammation. During his lifetime, he sired two litters. The testicles were collected immediately after noticing the death and arrived at the laboratory in 7 hours. The slicing of the epididymis caudae resulted in the collection of 1228.5×10^6 spermatozoa, of which 40% were motile. Normal morphology was observed in 33.5% of the sperm cells. All spermatozoa were subjected to cryopreservation according to protocol 1. After thawing, 32% of sperm cells remain viable, and post-thawing motility was 15%. Sperm morphology did not change (32.5%).

Case 10: fertility assessment and artificial insemination in an ocelot

A nine-year-old privately owned ocelot was kept together with a nine-year-old female. Copulations were noticed, but no kittens were born. The previous owners claimed that the male was fertile before. The collection of urethral semen allowed to obtain 95.5×10^6 spermatozoa of excellent motility (95%), suggesting that problems with fertility in this pair are not due to the poor quality of semen. The owner agreed to donate his ocelot semen to the Wrocław Semen Bank of Companion and Wild Animals - spermatozoa were cryopreserved according to protocol 1 (nine straws, 10×10^6 spermatozoa each).

The female ocelot was examined and, based on vaginal cytology, ultrasound, and blood analysis, follicular ovarian cysts and cystic endometrial hyperplasia were diagnosed. The female was treated with hCG (two injection of 400 IU, i.m, one day apart) and antibiotics (ciprofloxacin) for three weeks. Three months later, the owner requested artificial insemination. The female was hormonally stimulated by injection of 500 IU of eCG to induce estrus and ovulation was induced by injection of 250 IU of hCG 80 hours later [32]. Endoscopic transcervical artificial insemination was performed 40 hours after hCG injection. Semen was collected from the same male by urethral catheterization, resulting in the collection of 99.2×10^6 of spermatozoa, showing 95% motility. All spermatozoa were deposited in the uterus by transcervical intrauterine endoscopic insemination, but no pregnancy was obtained.

Case 11: postmortem semen collection in a Pallas Cat

A nine-year-old Pallas cat, kept in zoo, died in late November. Earlier this year (May) he fathered four kittens. Testicles were collected immediately after noticing the death of the animal and arrived at the laboratory in 12 hours. The slicing of the caudae of epididymides resulted in collection of few immotile spermatozoa. Considering the fact that the breeding season in this species lasts from December to April, the lack of spermatozoa was probably due to the nonreproductive time of the year.

Case 12: fertility assessment in two sand cats

Two sand cats: full brothers, twelve years old, were kept together in a zoo. They have never had a contact with female before. The zoo had the possibility to obtain a female for them, but due to the advanced age of both males (sand cats live up to 13-18 years in captivity), the zoo wanted to check whether they still produced spermatozoa. Semen was collected by urethral catheterization, followed by electroejaculation. In one male, no spermatozoa were obtained. The testicles of this male were small and had firm consistency. In the second male, 8.5×10^6 spermatozoa were collected by urethral catheterization (motility 50%) and additional 1.8×10^6 spermatozoa were collected by electroejaculation. The zoo agreed to donate sand cat semen to the Wrocław Semen Bank of Companion and Wild Animals. Two 0.125 ml straws, each containing 4×10^6 spermatozoa each, were cryopreserved according to protocol 1.

Regarding the fact that one of the males still produced semen, the zoo applied for a female. Sixteen months old female arrived one year later and after six months, one litter of 4 kittens was born (the father was a second male).

Case 13: semen collection from a serval on the occasion of anesthesia for veterinary purposes

A 12-year-old serval living in zoo was brought to the veterinary clinic for magnetic resonance examination (due to neurological symptoms). He sired multiple offspring, including three litters at a present location. The zoo requested to use the occasion of general anesthesia for neurological examination to collect and cryopreserve semen. By urethral catheterization, few, mostly immotile, spermatozoa were collected. No spermatozoa were collected by electroejaculation.

Case 14: fertility assessment in a serval

Serval, seven years old, was kept in zoo together with a female. Copulations were observed, but no offspring were obtained. Semen was collected by urethral catheterization, followed by electroejaculation, but no spermatozoa were obtained. The testes were small and atrophic. Regarding reproductive history and clinical findings, the male was considered infertile.

Case 15: artificial insemination in a serval

A private owner of a five-year-old serval asked for an artificial insemination because the male was not accepted for mating by a four-year-old female. At the same time, this male had three litters with another female. Semen was collected by urethral catheterization, followed by

electroejaculation. The first method resulted in the collection of 0.2 $x10^6$ spermatozoa (40% motility); no spermatozoa were obtained by electroejaculation. Insemination was abandoned due to an insufficient number of spermatozoa.

Case 16: fertility assessment in a snow leopard

A 10-year-old snow leopard, living in a zoo, sired two litters with two different females, but after that no offspring was obtained for next four years. Zoo asked to check the fertility of this male. Sperm was not obtained by urethral catheterization. In samples collected by electroejaculation, only a few non-motile sperm cells were observed. The male was considered most likely infertile. After semen examination, the male was moved to another zoo, where he was housed alone, so no further fertility could be evaluated.

Case 17: fertility assessment and artificial insemination in a tiger

A four-year-old tiger, living in zoo, was kept together with a six-year-old female. Attempts at copulations were noticed, but according to the zookeepers, they were not proper and no offspring was obtained. Zoo asked for artificial insemination. Due to the young age of the male (tigers reach puberty at the age of 4-5 years), the quality of semen was checked. Urethral catheterization did not allow to obtain any spermatozoa, however, by electroejaculation $120x10^6$ spermatozoa were collected, of acceptable motility (65%). The possibility of sperm collection from this male was confirmed, and the zoo agreed to cryopreserve the collected tiger semen. Twelve straws ($10x10^6$ spermatozoa each) were frozen according to protocol 1 and deposited in the Wrocław Semen Bank of Companion and Wild Animals.

Artificial insemination was performed one year later (no successful mating during that time). The female was hormonally prepared according to Donoghue et al. [34] (1000 IU of eCG, followed by 750 IU of hCG 80 hours later) and $100x10^6$ spermatozoa (60% motility) collected by electroejaculation were deposited in the uterus 45 hours after hCG using a transcervical endoscopic approach. The zookeepers reported behavioral changes and abdominal distention that could have been signs of gestation, but confirmation of pregnancy was not possible (no consent for anesthesia). There was no delivery at the expected time.
