# Supplementary material for: How Can We Introduce ART into Wild Felid Conservation in Practice? Joint Experience in Semen Collection from Captive Wild Felids in Europe
Source: Animals (Basel). 2022 Mar 30;12(7):871. doi: 10.3390/ani12070871 (PMC8997001; doi:10.3390/ani12070871)
Supplement: Supplementary file 1 [file animals-12-00871-s001.zip › Suppl Material Table S1.pdf]

Supplementary Material S1

Table S1. Anesthetic protocols used in the study:

Species	Anesthetic protocol
**Caracal** (***Caracal caracal***)	130 µg/kg medetomidine + 3.0 mg/kg ketamine
**Jaguar** (***Panthera onca***)	atropine 1.5 mg per animal + midazolam 0.15 mg/kg + xylazine 3.5 mg/kg + ketamine 3 mg/kg
**Leopard** ***(Panthera pardus)***	atropine 1.5 mg per animal + midazolam 0.15 mg/kg + xylazine 3.5 mg/kg + ketamine 3 mg/kg
**Ocelot** (***Leopardus pardalis***)	medetomidine 100 µg/kg + butorphanol 0.3 mg/kg
**Sand cat** (***Felis margarita)***	medetomidine 100 µg/kg
**Serval** ***(Leptailurus serval)***	1: medetomidine 75 µg/kg + butorphanol 0.1 mg/kg + midazolam 0.5 mg/kg<br>2: medetomidine 100 µg/kg + butorphanol 0.3 mg/kg, for electroejaculation additional injection of ketamine 2.5 mg/kg
**Tiger** (***Panthera tigris***)	medetomidine 80 µg/kg + tiletamine with zolazepam 1 mg/kg for electroejaculation additional injection of tiletamine with zolazepam 0.5 mg/kg

Cheetahs (*Acinonyx jubatus)* and snow leopard (*Panthera uncia*) were sedated by zoo veterinarian. Information about protocol were not collected.
